# Measuring school social climate in Latin America: the need for multidimensional and multi-informant tests – A systematic review

**DOI:** 10.3389/fpsyg.2023.1190432

**Published:** 2023-06-12

**Authors:** Mónica V. Bravo-Sanzana, Jorge Varela, Oscar Terán-Mendoza, Matias E. Rodriguez-Rivas

**Affiliations:** ^1^Núcleo Científico Tecnológico de Ciencias Sociales y Humanidades, Universidad de La Frontera, Temuco, Chile; ^2^Facultad de Psicología, Universidad del Desarrollo, Santiago, Chile; ^3^Doctorado en Psicología, Universidad de La Frontera, Temuco, Chile

**Keywords:** school social climate, Latin America, psychometry, systematic review, school climate, learning experiences, measurement

## Abstract

**Introduction:**

School social climate is central to understanding learning experiences in education environments. Previous studies describe various conceptual and operational definitions around the construct; however, there are no records of reviews focused on Latin America.

**Aim:**

This study sought to analyze the available evidence and the quality of school social climate measures in Latin America through a systematic review of the literature based on the PRISMA methodology and the COSMIN checklist to assess the psychometric properties of the instruments.

**Methodology:**

The Web of Science, Scopus, Psycinfo, and SciELO databases were consulted. A total of 582 records was identified, of which 27 fulfilled the inclusion criteria and methodological quality to be included in the systematization.

**Results:**

The results show that the country with the greatest scientific production on the topic is Chile, the measures are centered mainly on the students’ perspective and the most used instrument is the CECSCE. In addition, a common aspect to all the records is that they were not sufficient to capture the complexity of school social climate.

**Conclusion:**

Multidimensional and multi-informant measures are needed to adequately assess the construct.

## Introduction

School life is relevant and significant because interactions are produced there that generate a cultural mixture, given the family, regional, national, and institutional influences. The interactions generated can be conceived as school climate, refers to perceptions about the social, emotional and physical environment of a school, including the relationships between the different members of the educational community (attitudes and behaviors), as well as the norms, policies and practices that guide school behavior; it is a broad and multifaceted construct that encompasses factors such as safety, inclusion, academic expectations, and social support (Reyes et al., 2012; Claro, 2013; López de Mesa-Melo et al., 2013; Wang et al., 2018). This construct provides significant information on the experience of the student in the school, making it possible to know their perception of social relations in the school community, as well as the students’ motivation and commitment to this environment (Martínez-Ferrer et al., 2012).

This construct has also been referred to as school social climate (SSC), with special reference to the interactions generated in the school context and is defined as a dynamic element that arises from the perceptions of diverse actors in the education community on the relations they establish in this environment, at classroom and institutional level, which are developed according to the rules and behavioral habits established and regulated by each school (Trianes, 2000; Eccles and Roeser, 2011; Longobardi et al., 2022). It is a fundamental aspect of improving the equity and quality of education through learning to live with others, recognizing and valuing diversity. The SSC adheres to a social emphasis and to the role of each member of the educational community, teaching, and learning, representing a social right to form environments conducive to learning and promote the wellbeing of the community (Trianes et al., 2006; MINEDUC, 2019).

This is even more pertinent in the context of the pandemic (and post-pandemic), which has raised and will demand strong challenges to the school system in terms of education policies and sustainable programs to promote the wellbeing of the educational community (Vaillancourt et al., 2022). In this regard, while the importance of school climate is recognized, there is no consensus on the variables that comprise it or how to measure them (Wang and Degol, 2016; Grazia and Molinari, 2021). Reviews have been conducted worldwide showing this diversity; however, records on the study of the school climate focusing on Latin America are unknown.

### School climate as a construct

Historically, the study of climate in school contexts was focused on analyzing the classroom climate, which can be defined as the type of environment created by teachers and students, encompassing all the psychosocial dimensions of classroom life (Kurniawati and Hafina, 2020); from this two relevant approaches emerge, the first focused on those elements that favor the teaching-learning process (e.g. teacher quality, organization of the school environment) and those related to interpersonal relationships (e.g. coexistence between teachers and students and among peers (Wang and Degol, 2016). However, progress in the study of this construct revealed the need for a broader view that would incorporate other actors within the school environment and that would also allow the incorporation of other factors relevant to personal wellbeing, such as feelings of belonging (feeling accepted and valued by others) and structural elements such as the characteristics of the school environment (Trianes et al., 2006), from which the concept of SSC is derived. Nonetheless, it is necessary to differentiate school climate from classroom climate conceptually. The former is understood as a multidimensional construction that describes the perception that the members of a school community have with respect to the values, rules and thoughts that comprise it and of the relations that occur; however, classroom climate alludes to the perception that students and teachers have about those elements that influence the connections established in the classroom and the quality of learning (López et al., 2011).

Different studies show that there is no single definition of the school climate construct, nor is there a universal definition, but some authors opt for more concrete and conformable definitions, whereas others opt for an abstract and theoretical definition (Wang and Degol, 2016; Grazia and Molinari, 2021). In this perspective, the review by Cohen et al. (2009) defines SSC as the “quality and character of school life,” which are based on patterns of experiences (involving values, norms, pedagogical practices, organisation, among others) of school life that go beyond an individual experience, emphasising the group character of school life.

On the other hand, the review carried out by Thapa et al. (2013) point out that, “school climate – by definition – reflects students’, school personnel’s, and parents’ experiences of school life socially, emotionally, civically, and ethically as well as academically” (p. 369). Wang and Degol (2016) found that up to the date of their study there was a lack of consensus on the definition and, as such, the construct is used to refer to many different aspects of the school environment such as the beliefs, values and attitudes shared by members of the school community that shape interactions, behavioural parameters and school norms. Grazia and Molinari’s (2021) review synthesizes the definition by stating that this construct refers to individual perceptions of moral, relational and institutional aspects of school life. Likewise, and as a way of making a conceptual definition that identifies the components of the construct, Rudasill et al. (2018) propose the following definition: “school climate is composed of the affective and cognitive perceptions regarding social interactions, relationships, safety, values, and beliefs held by students, teachers, administrators, and staff within a school” (p. 46).

For the purposes of this review, SSC is defined as the perceptions of individuals regarding their personal relationships and intersubjective interactions, which are based on values and beliefs that therefore reflect a cognitive and affective elaboration. Likewise, the SSC incorporates aspects related to teaching-learning practices and organizational structure (classic components of classroom climate) that can contribute to the development of a favorable environment that fosters the integral development of individuals, because they feel physically, socially, and emotionally safe. Thus, the SSC arises from the perceptions of various actors in the educational community about the relationships they establish in this environment, at the classroom and institutional level, which are developed in accordance with the norms and behavioral habits established and regulated by each school.

It should be noted that even assuming this conceptual position, there is no universally accepted definition of SSC, however, a common point in the reviews made so far is the certainty that it is a multidimensional construct. For example, Cohen et al. (2009) performed a conceptual review of the literature, document analysis, and surveys (*n* = 40) based on actors related to the topic in order to bring together the key aspects of this construct. Thus, 4 large dimensions can be considered when referring to school climate: (i) Safety, physical and emotional, (ii) Teaching and instruction management, which may be oriented to strengthening positive relations among its members, (iii) A third dimension addresses relational aspects of the school members, both among peers and all the members of the school community, and (iv) Finally, those physical and environmental aspects of the school are considered because they are also relevant for a healthy coexistence. Later, Wang and Degol (2016), in another review of 327 different sources, propose another four dimensions (academic, community, safety, and institutional environment), confirmed by Grazia and Molinari (2021): academic environment, community environment, safe environment, and institutional environment.

For the purposes of this review, we adhere to the conceptual framework proposed by these studies by considering these four domains that incorporate the assumptions of classic conceptual frameworks such as Bronfenbrenner’s ecological model (Bronfenbrenner, 1987), Stage-Environment Fit Theory and Social Cognitive Theory.

### Measuring school climate

The measurement of school climate has been a challenge for different research teams considering the diversity of approaches and methodologies used for this purpose. As a first point, it can be mentioned that there are differences in the analysis of the data depending on whether they are oriented to understand the variable as an individual or institutional phenomenon, thus, some research has emphasized understanding the perception of school climate as a purely psychological variable based on individual perception, while other approaches at the institutional level propose that the idea of “climate” implies much more than the aggregation of individual scores, because, due to their psychosocial nature, they are nested at the school level and can explain other relevant variables within schools, such as levels of violence.

On the other hand, Cohen et al. (2009) emphasized in their findings two crucial aspects regarding the scientific study of school climate, the first is that the measures used were not focused equally on the three groups of central agents within educational environments (students, parents, and principals); the second is that the instruments are usually developed internally, which does not guarantee that they have the necessary methodological robustness, so that many of the measures could not be considered solid scientific tools. Also, evidence shows that different tools have been used to measure school climate, for example, disciplinary sanctions, teacher and staff retention, and student-teacher ratios which are considered as objective measures of the variable (O’Malley et al., 2012); However, given the strong influence of individual perception, these proxies have been considered inadequate and for this reason other types of measures that better capture subjectivity have been chosen, such as focus groups and questionnaires, which are not without their challenges given the need to include diverse perspectives, aiming at a multi-informant approach.

Previous studies make it possible to conclude that the operationalization of the construct of school climate continues to be as diversified as the quantitative instruments adopted to measure it, like surveys or validated measures. In the review by Wang and Degol (2016), based on 297 empirical studies, they show that approximately 92% of the examined works use the collection of survey data for school climate. The most used method is self-report surveys, and only 8% used qualitative methods, like discussion groups and interviews. These results are consistent with those obtained in later reviews. For example, Grazia and Molinari (2021), whose review included 113 articles between 2010 and 2018, showed that most studies were cross sectional, using self-report surveys on the personal perceptions of school climate at a single point in time. In addition, the authors reported that in more than 80% of the studies, the students were the only ones surveyed.

In Brazil, Lima and Peres (2022) conducted a scoping review of theses and dissertations published in that country starting in 1987, with a final sample of 70 studies. A predominance of qualitative research was observed (*n* = 34; 48.6%) over the quantitative (*n* = 25; 35.7%) and the mixed (*n* = 11; 15.7%). The most used collection methods were preexisting questionnaires and scales (*n* = 30; 42.9%) or developed by the authors themselves (*n* = 20; 28.6%) and interviews (*n* = 17; 24.3%). Students, teachers, and managers are the most studied groups. Generally, qualitative research tends to listen to different groups of subjects, whereas in quantitative research it is more common to listen to a single group, generally the students.

### Relevance of a new review for the Latin American context

The reviews conducted so far successfully achieve the conceptualization and identification of dimensions related to school social climate, however, they are not exempt from known limitations and biases when systematizing scientific evidence (Drucker et al., 2016); for example, the studies conducted by Cohen et al. (2009) and Thapa et al. (2013) are focused on documents related to public policies and school climate in the U.S. context that although they successfully identify dimensions of the construct and outcomes relevant to the study of school climate that could be partially generalizable, they address a local reality that does not necessarily represent the phenomenon in other countries; moreover, in none of these reviews was a search protocol reported that allows replicating the results obtained.

Regarding the review made by Wang and Degol (2016), it represents a valuable contribution to the conceptualization and state of the art in the study of school climate; however, in the manuscript it is not clear where the systematized evidence comes from. Something similar occurs with the review made by Grazia and Molinari (2021) who in their report inform a significant number of studies conducted in the United States and Europe, being notorious the lack of reports or instruments derived in the Latin American context. Likewise, both reviews systematize the measures used, but do not analyze them by means of tools to determine their psychometric quality and the identification of biases.

On the other hand, the report by Lima and Peres is a pioneer in systematizing evidence from the Latin American context and in accounting for the approaches used by different research groups in Brazil; however, this review does not adhere to the quality criteria for systematic reviews (PRISMA) and focuses specifically on a Brazilian context.

It is also necessary to highlight that in Spanish-speaking countries, a concept that has been used as a synonym for SSC is that of school coexistence, since it refers to a perspective in which interpersonal relationships are fostered whose quality is positive and counteracts the phenomena of violence that occur in school environments. For Del Rey et al. (2009) learning to live together and feeling that the school is a safe and satisfactory place is related to perceiving that there is a good coexistence in the school, therefore, it is common for those investigations that measure the effect of strategies to favor an SSC to refer to the concept of coexistence, because this is considered an institutional management tool.

In this sense, it is necessary to examine in depth which factors or dimensions are included within the instruments in the Latin American context, as it would then be possible to determine their relevance in predicting outcomes in health and wellbeing. In addition, the trend in the study of school climate is oriented to obtaining measures where the informants are usually the children and adolescents, omitting other sources of intelligence like the school’s teachers and administrative personnel, which would contribute relevant and significant indicators for the measurement of the construct given its multidimensional and multilevel nature (Chin et al., 2008). This is high priority considering that school climate has effects on areas of the students’ lives such as better academic performance (Thapa et al., 2013), higher levels of school motivation (Eccles et al., 1993), fewer mental health issues (Varela et al., 2021a), and wellbeing over time (Benbenishty and Astor, 2005; Suldo et al., 2013; Varela et al., 2021b).

### Aim of the study

Thus, previous studies have demonstrated the poor use of multiple informants in the studies, confirming that the great majority of the studies were based on a measure performed on the students, and that an only a small number of studies had the participation of teachers and very few articles considered the parents’ perceptions. In addition, in the case of systematic reviews conducted in an English-speaking context, there is coverage bias since they do not include databases specialized in Latin American scientific production (e.g., ScieLO), or terminology commonly used in texts written in Spanish like “coexistence,” which has been used as synonymous for school climate in studies in Latin America, is not incorporated. This brings with it two limitations, the first is the lack of knowledge of the cultural elements of the different Latin American contexts, which would undoubtedly allow a complete conceptualization of the construct of school social climate, as a consequence, a second limitation is that the measures developed could be insufficient to address a school reality in which schools play a protective role for children and adolescents, especially in those contexts characterized by a high rate of violence and social vulnerability. Consequently, the aim of the present systematic review is to identify and evaluate the existing instruments to measure the perception of school climate in Latin America and evaluate the quality of their psychometric properties.

## Methodology

### Design and search strategy

The protocol of the present review is registered in the PROSPERO platform under the ID CRD42023392569 and was performed following the PRISMA (Preferred Reporting Items for Systematic Reviews and Meta-analyses) guidelines and recommendations (Moher et al., 2015; Page et al., 2021). The article search took place from May to July 2022, through the Scopus, Web of Science (WOS), ScieLO and PsycINFO databases, including articles published since 2010.

The search strategy included the following key terms: [“School social climate” OR “School life” OR “Schoolwide climate” OR “School environment” OR “school climate” OR “school coexistence” OR “educational community” OR “classroom climate” OR “school connivance”] AND [Scale OR Measure OR Measuring OR Score OR Rating OR Survey OR Questionnaire OR Inventory OR Index OR Evaluation OR Assess* OR Instrument OR measurement OR detection OR diagnos* OR checklist OR psychometr* OR reliability OR validity] AND [Argentina OR Bolivia OR Chile OR Colombia OR “Costa Rica” OR Cuba OR “Dominican Republic” OR Ecuador OR “El Salvador” OR Guatemala OR Honduras OR Mexico OR Nicaragua OR Panama OR Paraguay OR Peru OR “Puerto Rico” OR Uruguay OR Venezuela OR “Hispanic America” OR Caribbean OR “Central America” OR “South America” OR “Latin America” OR “Spanish-speaking population”]. Additionally, a manual article search was done in August 2022 to identify recently published literature, by checking the reference lists of relevant studies. Specific syntaxis for each data base is available in Supplementary material.

### Inclusion and exclusion criteria

Published quantitative articles in English, Spanish, or Portuguese performed on a Latin American population, which included the measurement of the school climate, incorporated some of the following participants: adolescent students, parents and guardians, principals/institutional management group, administrative personnel, and/or teaching assistants, were included. The studies not performed on a population in Latin American countries, samples comprised of preschool, kindergarten, primary or university students were excluded, as were qualitative studies when analysis of their psychometric properties was not possible.

### Article selection

After eliminating duplicate articles through bibliographical reference software Endnote, two independent investigators (MER-R and OET-M) selected articles for inclusion using the online version of the Rayyan tool (Ouzzani et al., 2016). A third investigator (MBS) examined all the articles included for this selection and resolved any discrepancies in the decision criteria, through a rigorous and critic review of these articles. The eligibility of the search results was examined in two stages: first by title and abstract, and then in full text. The reasons for exclusion for each discarded manuscript were recorded, using the preset labels available on the Rayyan platform so as to offer greater specificity for the reasons for exclusion (e.g., wrong publication when it the publications differed from reviewed articles).

In addition, to check the degree of agreement in the study selection and coding phases, inter-coder reliability was calculated using Cohen’s *κ* coefficient, which had a value of 0.71, indicating substantial inter-coder agreement.

### Data extraction and evaluation of methodological quality

For the selection phase by title and abstract, both reviewers used an analysis guideline based on the inclusion and exclusion criteria along with the key words to guarantee precision in the selection. Next, the data were extracted independently through a data extraction form, which specified the relevant aspects of the publications including the country, name of the instrument, sample size, informant type, domains of the school climate measure and characteristics of the scale.

Additionally, to assess the methodological quality of each instrument and its psychometric properties, two independent investigators (MER-R and OET-M) used a checklist based on COnsensus-based Standards for the selection of health Measurement INstruments (COSMIN) (Terwee et al., 2012), through which various quality criteria were evaluated on a scale of four points (very good, adequate, doubtful, inadequate). The present systematic review included in the evaluation the following sections of the COSMIN Risk of Bias checklist (Mokkink et al., 2018): (i) content validity, (ii) structural validity, (iii) internal consistency, (iv) transcultural validity, (v) test–retest, and (vi) construct validity. Finally, the 2 independent data extraction reviews were compared and the articles that did not have agreement were discussed with a third reviewer (MBS), through detailed analysis in each of these articles.

## Results

**Figure 1:**
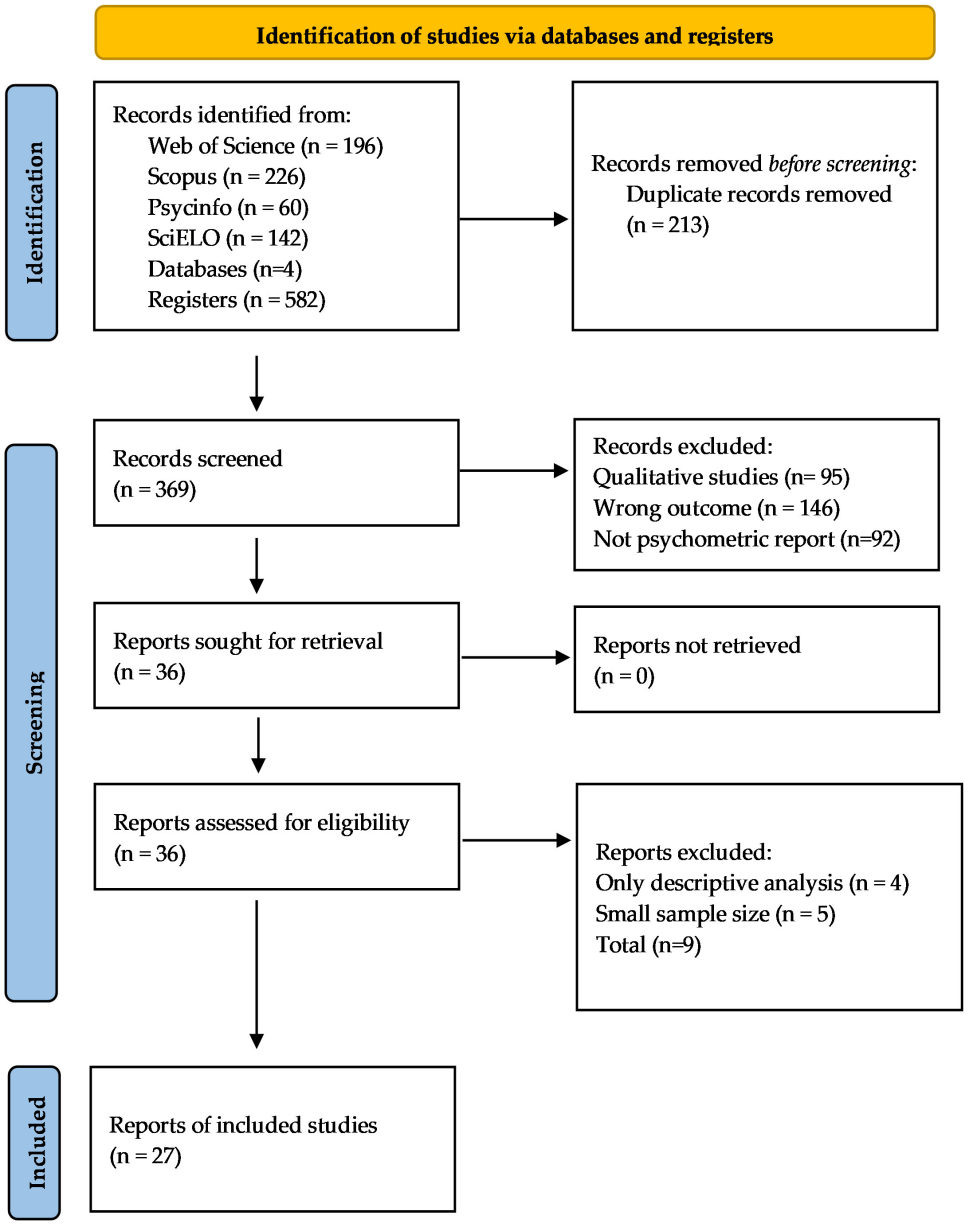
PRISMA flow diagram of the systematic review process.

The strategy search resulted in 582 references from the different databases; after eliminating the duplicate records there were 369 records and the article filtrate was carried out, leaving a total of 27 scientific articles eligible for systematization. The specifications inherent to the reasons for discarding appear in Figure 1. Specifically, from the screened records, 333 articles were eliminated because they were only qualitative articles (*n* = 95), had wrong outcomes (not related to school climate or its denominations) (*n* = 146), or the analysis of their psychometric properties was not possible (*n* = 92). Finally, of the 36 assessed for eligibility articles, 9 articles were eliminated because they only reported descriptive analyses (*n* = 4), or the total sample sizes were insufficient (*n* = 5).

### Bibliometrics data

From the 27 analyzed reports, 51.9% (*n* = 14) are from Chile, followed by Mexico with 19.2% (*n* = 5), studies with mixed samples that included two countries 19.2% (*n* = 5), Colombia with 7.7% (*n* = 2) and the Dominican Republic 3.8% (*n* = 1). The transcultural studies tested invariance in instruments from Spain with Costa Rica (*n* = 2), Chile with Colombia (*n* = 1), Chile with Spain (*n* = 1), and the United States with Mexico (*n* = 1). With respect to the informants who completed the instruments in the studies, the vast majority were only students (*n* = 22, 84.6%); to a lesser extent analyses based on teachers’ responses (*n* = 2; 3.9%) and mixed samples of students and teachers (*n* = 2) or school personnel and parents (*n* = 1) were reported.

### Dimensions of the measures

As shown in Table 1, the type and number of domains of the included articles were analyzed, according to the four-domain classification of Wang and Degol (2016). Specifically, it was observed that all the articles included the community domain, 93% the safety domain, 85% the academic domain and only 30% the institutional environment domain. Table 2 shows in detail the characteristics of the 27 studies included in this review. Special emphasis is placed on the dimensions that make up each instrument along with the characteristics of the scale and the samples in which they were used.

**Table 1:** Types and number of school climate domains of the included studies according to Wang and Degol’s (2016) classification (*n* = 27).

	*n*	%
Domains		
Safety	25	93
Community	27	100
Academic	23	85
Institutional environment	8	30

**Table 2:** Summary of the main characteristics of each study.

Authors [Countries]	Name of the instrument	Sample characteristics	Dimensions/factors	Psychometric index
Alonso-Tapia et al. (2019) [Spain and Costa Rica]	Group co-living climate questionnaire – GCCQ Student’s contribution to co-living climate – SCCCQ	Secondary students *n* = 2,581 (2038 in Costa Rica and 543 in Spain)	Classroom co-living climate; Praise others’ achievements sincerely; Active listening; Trying to understand their peers’ points of view; Helping others; Stand up for a peer; Pay emotional attention to peers to avoid that they remain isolated; Prevent anyone from feeling imprisoned in the group.	*ω* = 0.78–0.85 Confirmatory factor analysis. Strong invariance for GCCQ and weak invariance for SCCCQ between countries samples. Discriminant validity with social integration measure.
Alonso-Tapia et al. (2020) [Spain and Costa Rica]	The School Climate Battery of Questionnaires for Secondary and High School Teachers (SCBQSHST)	Secondary and baccalaureate teachers *n* = 521 (178 in Costa Rica and 343 in Spain)	Quality of leadership; Quality of support among teachers; Learning motivational orientation; Performance motivational orientation; Quality of student attitudes; Parental support	*ω* = 0.83–0.99 Confirmatory factor analysis. Strong invariance for Quality of support among teachers and Quality of student attitudes. Non-invariance for others sub-scales between countries samples. Convergent validity with satisfaction measures.
Aron et al. (2012) [Chile]	School social climate scale (ECLIS)	Students *n* = 300	Relations Teacher–students; Physical training conditions; Relations between pairs; General evaluation of the school; Bullying	*α* = 0.63–0.89 Exploratory factor analysis. Construct validity trough inter-scales correlations.
Bravo-Sanzana et al. (2019) [Chile]	Questionnaire to evaluate School Social Climate (CECSCE)	Secondary students *n* = 2,352	School social climate (CSE) Social climate of the teachers (CSP)	*ω* = 0.76–0.82 Confirmatory factor analysis. Strict invariance between men and women. Bivariate analysis between groups.
Bravo-Sanzana et al. (2020) [Chile]	The Student Context Questionnaires from the SIMCE 2015 (CCES2015)	Secondary students 158,572	Promotion of participation in class; Climate in the classroom; Climate of trust in the school; Discrimination; School violence; Student–teacher violence; Student safety; Bullying; Disciplinary measures; Illicit actions in school; Participation in school activities; Leadership in school activities; Satisfaction with the school; Identity with the school	*ω* = 0.66–0.94 Exploratory and confirmatory factor analysis. Strict invariance between men and women.
Bringas-Molleda et al. (2021) [Dominican Republic]	M-EP Scale	Students *n* = 1945	Values Education and Conflict Resolution; Consensus of Classroom Rules; Dissemination of Rules and Penalties to Family	*α* = 0.57–0.79 Confirmatory factor analysis. Bivariate analysis between sub-samples urban and rural.
Caso et al. (2014) [México]	School Coexistence Questionnaire	Students *n* = 1,259	Inclusive coexistence; Democratic coexistence; Peaceful coexistence	*α* = 0.92–0.97. Alfa if item is dropped. Discrimination analysis of items. Homogeneity index.
Chaparro et al. (2015) [México]	School Coexistence Questionnaire	Students *n* = 1,254	Inclusive coexistence; Democratic coexistence; Peaceful coexistence	*α* = 0.63–0.94 Exploratory factor analysis.
Cruz Flores (2021) [México]	Inclusive Cultures in Secondary and Higher Education Scale (EMS)	Students *n* = 1,200	Responsibility with learning and sense of belonging; Affective school environment; Social construction of knowledge; Development of citizenship and social participation; School safety and its environment	*α* = 0.63–0.86 Principal components analysis.
Gálvez Nieto et al. (2014) [Chile]	Questionnaire to evaluate School Social Climate (CECSCE)	Students *n* = 977	School social climate (CSE) Social climate of the teachers (CSP)	*α* = 0.70–0.84 Exploratory factor analysis. Convergent validity through correlations with measures of victimization and violent delinquency behavior.
Gálvez-Nieto et al. (2015) [Chile]	Questionnaire to evaluate School Social Climate (CECSCE)	Students *n* = 2,306	School social climate (CSE) Social climate of the teachers (CSP)	*α* = 0.73–0.80 Exploratory and confirmatory factor analysis. Test–retest analysis. Convergent validity through correlations with measures of attitude to authority and attitude to transgression.
Gálvez-Nieto et al. (2016) [Chile and Colombia]	Questionnaire to evaluate School Social Climate (CECSCE)	Students *n* = 1,223 (692 Chilean students and 541 Colombians students)	School social climate (CSE) Social climate of the teachers (CSP)	*α* = 0.58–0.81 Confirmatory factor analysis. Weak invariance between countries samples.
Gálvez-Nieto et al. (2020) [Chile]	School Climate and School Identification Measure-Student Scale (SCASIM-St)	Secondary students *n* = 1,456	School Climate (4 factors: Student–Student Relations; Student–Staff Relations; Academic Emphasis and Shared Values and Approach); School Identification (1 factor: School Identification)	*ω* = 0.84–0.92 GLB = 0.85–0.93 Confirmatory factor analysis. Convergent validity through correlations with impartiality of school discipline and student support measures.
Gálvez-Nieto et al. (2021a) [Chile]	Authoritative School Climate Survey (ASCS)	Students *n* = 808	Disciplinary Structure; Student Support	*ω* = 0.73–0.84 Exploratory and confirmatory factor analysis. Convergent validity through correlations with attitude to authority and attitude to transgression measures.
Gálvez-Nieto et al. (2021b) [Chile]	Abbreviated Version of the Dual School Climate and School Identification Measure-Student (SCASIM-St15)	Students *n* = 2044	School Climate (4 factors: Student–Student Relations; Student–Staff Relations; Academic Emphasis and Shared Values and Approach); School Identification (1 factor: School Identification)	*ω* = 0.73–0.84 Confirmatory factor analysis. Strong invariance for education level and age and weak invariance by gender. Convergent validity through correlations with attitude to authority and attitude to transgression measures.
Guerra Vio et al. (2011) [Chile]	Questionnaire to evaluate School Social Climate (CECSCE)	Students *n* = 1,075	School social climate (CSE) Social climate of the teachers (CSP)	*α* = 0.72–0.83 Exploratory factor analysis Convergent validity through correlations with a school violence measure.
Higuita-Gutiérrez and Cardona-Arias (2016) [Colombia]	Questionnaire of School Climate and Safety (CSCSS) [California School Climate and Safety Survey]	Students *n* = 3,406	Unsafe behaviors; School climate; Unsafe conditions; Victimization	*α* = 0.81–0.91 Principal components analysis. Bivariate analysis by living area and gender.
Larios Gómez (2021) [México]	Not reported	Teachers and students (*n* = 215 teachers and *n* = 150 students)	For teachers: Perceived Classroom Climate; Perceived School Climate. For students: Perceived Classroom Climate; Student’s self-concept; Rules of coexistence	*α* = 0.81–0.91 Correlations between items. Sphericity Bartlett test. ANOVA between groups.
López et al. (2014) [Chile]	School Climate scale of the Instrument for Students created by Khoury-Kassabri	Students *n* = 4,688	Social support; Rules; Rules violence; Participation	*α* = 0.89 Exploratory and confirmatory factor analysis. Multivariate test of differences by gender and school establishment.
López and Valdés Morales (2018) [Chile]	Not reported	Fathers and Education professionals (*n* = 1,129 fathers and *n* = 741 professionals)	Inclusive culture; Inclusive practices; Further education; Team-work orientation; Shared rules; Democratic Participation; Punitive practices; Willingness to change; Organizational willingness; Culture of improvement of coexistence; Prevention of coexistence problems; Promotion of good coexistence	*α* = 0.72–0.92 Confirmatory factor analysis. Convergent validity through correlations with number of violence and discrimination reports filed
López et al. (2018) [Chile]	Not reported	Students *n* = 6,813	Peer Relationships; Teacher-Student Interaction; Physical environment; Teacher’s orientation towards learning	*α* = 0.75–0.86 Confirmatory factor analysis. Convergent validity through correlations with a peer victimization measure.
Muñoz et al. (2018) [Chile and Spain]	School-wide Climate Scale (SCS)	Students (1,169 Chilean and 1,278 Spanish)	Positive Interpersonal Management; Victimization; Disruptiveness; Peer social network; Aggression; Normative adjustment; Indiscipline; Teacher apathy	*α* = 0.72–0.89 Confirmatory factor analysis. Strong invariance by gender in Chilean sample and between countries.
Muñoz-Troncoso et al. (2017) [Chile]	Questionnaire of School Coexistence for Non-violence (CENVI)	Students *n* = 1,410	Factor 1. Types of School Violence include five dimensions: Verbal violence; Physical-Behavioral violence; Social-Exclusion violence; Violence Technological Media; Teacher to Student Violence Factor 2. Management of Coexistence include three dimensions: Non-violence training; Non-violence management; Participation	*α* = 0.85 and 0.90 Confirmatory factor analysis
Román-Calderón et al. (2017) [Colombia]	School Environment Survey	Teachers *n* = 3,610	Teacher academic expectations; Communication; Engagement; Safety and respect	*α* = 0.56–0.85 Multilevel exploratory factor analysis.
Shukla et al. (2019) [Mexico and Unites States]	Maryland Safe and Supportive Schools (MDS3) School Climate Survey	Students (15,099 from United States and 2,211 students from Mexico)	Evaluates 3 dimensions and 13 sub-scales: Student engagement (6 sub-scales): Teacher Connectedness; Student Connectedness; Student Achievement; Whole-School Connectedness; Culture of Equity; Parent Involvement Safety (3 sub scales): Bullying; Physical Safety; General Drug Use School environment (4 sub-scales): School Rules and Consequences; Physical Comfort; Support; Disorder	*α* = 0.51–0.90 Strong invariance for engagement and safety. Weak invariance for environment.
Tapia-Fondle et al. (2020) [México]	Not reported	Students *n* = 405	Evaluates three dimensions and 10 sub-scales: Physical (3 sub-scales): Classroom; Schoolyard; Libraries Social (3 sub-scales): Justice; Sustainability; Social coexistence Academic (4 sub-scales): Teacher’s relationship with student; Teaching methods; Evaluation; Didactic strategies	*α* = 0.47–0.90 Confirmatory factor analysis. Convergent validity trough correlation with a wellbeing measure.
Valdés Morales et al. (2018) [Chile]	Questionnaire of school coexistence in its reduced version	Students *n* = 2,868	Inclusive coexistence; Democratic coexistence; Peaceful coexistence	*α* = 0.87–0.95 Confirmatory factor analysis.

#### School coexistence

A subgroup of instruments has focused specifically on addressing the construct of coexistence in the school environment. This points directly to the development of a pleasant environment oriented toward social relations based on respect and wellbeing that has been conceived as synonymous with school climate in the Latin American literature. There are instruments like the CENVI (Troncoso et al., 2017) that assess sources of violence and the actions to encourage coexistence, a conceptualization similar to that of the SCS (Muñoz et al., 2018) with the difference that the latter does not include action to promote coexistence but sources of social support in the school environment. On the other hand, the M-EP questionnaire (Bringas-Molleda et al., 2021) concentrates on the students’ perception of measures that improve the school coexistence specifically focused on conflict resolution and institutional regulations, the items of which refer directly to the actions taken by managers, teachers, a measure similar to that used by Valdés Morales et al. (2018), for being based on actions to improve coexistence in the school environment, with the reservation that the latter is directly centered on the report of parents and managers of the schools. In addition, one of the analyzed instruments is focused directly on measuring perception of the effectiveness of the coexistence measures by addressing bullying (Higuita-Gutiérrez and Cardona-Arias, 2017).

An instrument that has three versions reported in the studies concentrates on analyzing the actions of teachers, but based on three dimensions called democratic, peaceful and inclusive (Caso Niebla, 2013; Chaparro et al., 2015; Valdés Morales et al., 2018). In the same vein, the proposed measure by de la Cruz Flores (2021) focuses on the measure of inclusive behaviors; however, it is important to highlight that the author refers to the term inclusive school culture, within which school climate is used a subcomponent. Finally, the GCCQ (Alonso-Tapia et al., 2019) evaluates the perception regarding the actions of classmates and one’s own actions to promote school coexistence and starts from the perspective of interactions among students beyond the connection with the teacher. It is an instrument that is answered in a dual perspective from the contribution of the school group and from one’s own actions to promote coexistence and is based on a behavioral domain as they are grouped as a series of strategies.

#### School climate

Studies based on teachers like that by Alonso-Tapia et al. (2020) focuses on aspects related both to the exercise of the role of teacher (e.g., leadership, quality of support offered to the students, and satisfaction with teaching) and to the perception of certain elements inherent to the participants of the process like the students (e.g., attitudes), the parents (e.g., quality of support), and to the school philosophy, analyzed in particular from motivation.

There are also questionnaires that focus on addressing the phenomenon from a perspective of the students referring to different levels, for example the ECLIS (Aron et al., 2012), on the relations with peers, physical conditions of the school facilities, relations with the teachers and satisfaction with the school. These last three dimensions are also operationalized by two other instruments. The first widely reported for its brevity and consistency in factorial analyses is the CECSCE (Guerra Vio et al., 2011; Gálvez Nieto et al., 2014; Gálvez-Nieto et al., 2015, 2017; Bravo-Sanzana et al., 2019), made up of two dimensions: one that explores the students’ perception of their teachers and a second called school climate which is made up of items that refer to aspects of the physical environment such as the feeling of security, respect and safety within the school grounds. On the other hand, the scale developed by López et al. (2018) also includes relations with peers, teachers and the physical environment, a dimension of learning-oriented teaching. Another brief instrument used in the reported studies is the ASCS, which is made up of the factors of disciplinary structure and support for the students; the particularity of this measure is that the construct corresponds to an authoritarian school climate (Gálvez-Nieto et al., 2021a).

In this order of ideas, from Bronfenbrenner’s ecological approach (Bronfenbrenner, 1987), the existence of different spheres that make up school climate similar to those previously mentioned is proposed, but in addition it involves a subcomponent of identification with the school; this dual operationalization is adopted by the SCASIM questionnaire in its version for students that has psychometric analyses for the long version (Gálvez-Nieto et al., 2020) and for the brief one (Gálvez-Nieto et al., 2021b). Identification with the school is also included as a subcomponent of school climate in the analysis performed from indicators on the SIMCE by Bravo-Sanzana et al. (2020) under the concept of SSC, which incorporates several components of the scales that report clearly measuring school climate as one of the measures of coexistence. Other studies that point to the integration of conceptual aspects related to coexistence and SSC are the School Climate Scale by López and Valdés Morales (2018) that deconstructs school climate into regulations, social support and participation, and the coexistence management measure by Larios Gómez (2021) that explores school climate and classroom climate for teachers and students. Finally, some reports operationalized the analysis of the construct from the school environment (Román-Calderón et al., 2017; Shukla et al., 2019; Tapia-Fonllem et al., 2020) having as common elements aspects related to safety/physical comfort, commitment and support networks of teachers and students.

#### Psychometric quality of the instruments

Finally, in relation to the quality of the instruments, the assessment of each of their sections can be seen in Table 3. In this regard, the two properties reported in both studies were internal consistency and the factorial structure analysis, whereas the least reported property was the temporal stability analysis, which was only reported in one study and its quality was considered inadequate given the small sample size for that specific analysis. Moreover, although it was an aspect of validity explored in many of the articles, the content validity was inadequate in most of them because no report was made of the characteristics of the expert judges who perform the review, the criteria on which this evaluation was based nor how discrepancies in the translation processes of scales were resolved.

**Table 3:** Evaluation of the psychometric properties adapted from COSMIN criteria.

Study	Content	Structure	Internal consistency	Invariance	Test–retest	Construct
Alonso-Tapia et al. (2019)	Doubtful	Very good	Very good	Adequate	–	Very good
Alonso-Tapia et al. (2020)	–	Very good	Very good	Adequate	–	Very good
Aron et al. (2012)	Doubtful	Inadequate	Very good	–	–	Inadequate
Bravo-Sanzana et al. (2019)	–	Very good	Very good	Adequate	–	–
Bravo-Sanzana et al. (2020)	–	Very good	Very good	Adequate	–	–
Bringas-Molleda et al. (2021)	Inadequate	Very good	Doubtful	–	–	Very good
Caso et al. (2014)	–	Inadequate	Very good	–	–	–
Chaparro et al. (2015)	Doubtful	Adequate	Very good	–	–	–
Cruz Flores (2021)	Adequate	Adequate	Very good	–	–	–
Gálvez-Nieto et al. (2014)	Doubtful	Doubtful	Very good	–	–	Very good
Gálvez-Nieto et al. (2015)	–	Very good	Very good	–	Inadequate	Very good
Gálvez-Nieto et al. (2016)	Doubtful	Very good	Doubtful	Very good	–	–
Gálvez-Nieto et al. (2020)	Doubtful	Very good	Very good	–	–	Very good
Gálvez-Nieto et al. (2021a)	Doubtful	Very good	Very good	–	–	Very good
Gálvez-Nieto et al. (2021b)	–	Very good	Very good	Adequate	–	Very good
Guerra Vio et al. (2011)	Doubtful	Very good	Very good	–	–	Very good
Higuita-Gutiérrez and Cardona-Arias (2016)	–	Doubtful	Very good	–	–	Inadequate
Lario-Gómez (2021)	Doubtful	Inadequate	Very good	–	–	Inadequate
López et al. (2014)	Doubtful	Very good	Very good	–	–	Very good
López and Valdés (2018	Adequate	Very good	Very good	–	–	Very good
López et al. (2018)	Very good	Very good	Very good	–	–	Very good
Muñoz et al. (2018)	Inadequate	Very good	Very good	Very good	–	–
Muñoz-Troncoso et al. (2017)	Doubtful	Very good	Very good	–	–	–
Román-Calderón et al. (2017)	Inadequate	Adequate	Adequate	–	–	–
Shukla et al. (2019)	Doubtful	Very good	Doubtful	Adequate	–	–
Tapia-Fondle et al. (2020)	–	Adequate	Doubtful	–	–	Very good
Valdés et al. (2018)	Inadequate	Very good	Very good	–	–	–

Another relevant point is the evidence of criterion validity of the systematized instruments through their relationships with other variables: in the case of the measures focused on coexistence, they were inversely related to indicators associated with violence in the school context and bullying (López et al., 2014; Bringas-Molleda et al., 2021); this would end up being a noteworthy element when establishing strategies to favor the quality of relationships in the educational context; likewise, the measures focused directly on SSC showed positive correlations with aspects related to social integration (Alonso-Tapia et al., 2019), satisfaction and motivation (Alonso-Tapia et al., 2020) positive attitudes towards authority (Gálvez-Nieto et al., 2015, 2021a,b), student support (Gálvez-Nieto et al., 2020) and wellbeing (Tapia-Fonllem et al., 2020), negative correlations with criminal conduct (Gálvez Nieto et al., 2014), and scholar violence (Guerra Vio et al., 2011).

## Discussion

This study sought to analyze the scientific evidence available regarding psychometric measures of school climate in Latin America. The review gives an account of the diversity of dimensions measured when evaluating school climate. Some focus solely on school coexistence and others examine different dimensions of climate, which reveals the theoretical diversity demonstrated in Latin America. In this perspective, school space becomes relevant and significant because in it interactions are produced that generate a cultural mix, given the family, regional, national and institutional influences (Trianes et al., 2006; MINEDUC, 2019), which arises from the perceptions of diverse actors in the education community on the relations they establish in this environment, at classroom and institutional level, which are developed according to the rules and behavioral habits established and regulated by each school (Claro, 2013; López de Mesa-Melo et al., 2013).

The evidence collected shows that the most widely used instrument, the CECSCE, presents adequate psychometric properties of internal consistency invariance by sex and between countries, specifically Colombia and Chile, it also shows construct validity through positive correlations with measures of attitudes towards authority and negative correlations with instruments that measure transgression, violence at school, victimization and attitudes towards violence and delinquency; however, conceptually it is not able to satisfactorily address the complexity of SSC as a construct, since only the aspects related to interpersonal relationships and the physical environment of the center predominate, leaving aside the academic and safety elements of the center proposed both by Cohen et al. (2009) and by Wang and Degol (2016). Something similar occurs with the measures focused on coexistence, given that by nature their approach would be centered on physical and emotional safety in addition to social relations, which is why these measures do not incorporate academic or structural aspects related to the physical environment. Even so, it is worth noting two instruments that are much closer to a satisfactory multidimensional measure (Shukla et al., 2019; Tapia-Fonllem et al., 2020), given that they have multiple subscales that favor obtaining information from various domains necessary for understanding the SSC, with the caveat that these were developed solely from the perspective of the students.

Regarding the relationship of these instruments with other variables, there is a tendency to analyze climate measures from their capacity to show a relationship with variables related to the harmony of interpersonal relationships, especially with the reduction of violence indicators and student attitudes towards authority figures in the school environment. It is striking that at least in the studies to develop the measures, there is no approach oriented to analyze classical variables in this area such as academic performance (Thapa et al., 2013), motivation, or even variables related to mental health (e.g., anxiety and depression) (López et al., 2014; Varela et al., 2019), which is probably because, as already mentioned, systematized studies do not usually include these indicators, which have been classically associated with what is known as classroom climate.

Considering that climate measures are related to the reduction of acts of violence in schools, it would be necessary for them to simultaneously incorporate the perspective of other agents within the school such as teachers and management personnel, considering that these would be in charge of developing management strategies in the schools and their perception ends up being relevant at the moment of identifying aspects sensitive to the intervention that favor positive results (Grazia and Molinari, 2021), and only one of the studies (Alonso-Tapia et al., 2020) analyzes the teachers’ perspective related to these outcomes. It is also necessary for those researchers interested in the development of SSC measures to show through psychometric analysis their relationship with academic outcomes, which although they have been tested in other empirical studies, the evidence gathered in this review shows that it has not been considered as a relevant element in the development of psychometric instruments.

Likewise, there were adequate reports of reliability, but unlike previous reviews, there were no analyses that guaranteed the temporal stability of the measurements; the only study focused on this methodology had an insufficient sample size, which makes it impossible to guarantee the quality of the results; therefore, future studies should aim to perform analyses of temporal invariance. This would make it possible to guarantee that the results obtained in the instruments are not exclusively due to transitory elements associated with measurement error and that the scores truly respond to the latent variable to be measured (Widaman et al., 2010).

In addition, from the multidimensional and multilevel perspective, to understand SSC it is necessary to incorporate elements related to school coexistence, which according to various authors refers to the management instruments related to legislative and planning policies to create an environment that is satisfactory for knowledge management (López et al., 2018). From this point of view, the assessment by the different actors involved (students, teachers, parents, and managers) regarding actions to promote harmonious environments becomes an element to contemplate when measuring school climate because it is a component that ends up shaping a proportion of the general assessment on school climate.

Although the strengths of this review were its development under the PRISMA methodology and the evaluation of the psychometric quality of the instruments through standardized criteria such as COSMIN, some limitations can also be mentioned, the first of which is that unpublished articles were not included in the review, thus increasing the risk of publication bias. In addition, some of the articles come from the same research groups or were conducted on the same study samples, which may lead to an underrepresentation of the populations studied in the included articles.

## Conclusion

The present review gives an account of the existence of scales in Latin America in different countries, but mainly in Chile, using students as the main source of information. This represents a challenge for future measurements of school climate, since it is a collective perception of the members of a school community, and at the same time, it requires that the data be analyzed hierarchically. This involves the of multivariate techniques, which combine the confirmatory factor analysis and structural equation modeling with a multilevel approach needed to reflect a more precise measurement of the characteristics of the school. The argument is based on the standard use of a single scale to represent an aspect of the climate not sufficiently controlling for measurement error, and from there the need to construct models with latent constructs that represent the climate for the individual and for the environment (Grazia and Molinari, 2021).

In conclusion, the present review reveals the diversity of components that forms the climate school measures in psychometric studies conducted in Latin America, and like previous reports, there is evidence that although some of the instruments used have suitable psychometric properties, they do not ultimately address the complexity that the school climate implies. First of all, because they only address some of its components and, in addition, because they mainly take students as their only source of intelligence, which although they have a role in the education environment, they are not the only ones responsible for the construction of school climate.

## Author contributions

MB-S and JV: conceptualization, validation, investigation, and supervision. OT-M and MR-R: methodology, formal analysis, and data curation. MB-S: resources, project administration, and funding acquisition. MB-S, JV, OT-M, and MR-R: writing—original draft preparation, writing—review and editing, and visualization. All authors contributed to the article and approved the submitted version.

## Funding

This work was funded by the ANID, FONDECYT REGULAR 1220166.

## Conflict of interest

The authors declare that the research was conducted in the absence of any commercial or financial relationships that could be construed as a potential conflict of interest.

## Publisher’s note

All claims expressed in this article are solely those of the authors and do not necessarily represent those of their affiliated organizations, or those of the publisher, the editors and the reviewers. Any product that may be evaluated in this article, or claim that may be made by its manufacturer, is not guaranteed or endorsed by the publisher.
