# Supplementary material for: Measuring school social climate in Latin America: the need for multidimensional and multi-informant tests – A systematic review
Source: Front Psychol. 2023 Jun 12;14:1190432. doi: 10.3389/fpsyg.2023.1190432 (PMC10316712; doi:10.3389/fpsyg.2023.1190432)
Supplement: Supplementary file 1 [file Data_Sheet_1.docx]

Suplemmentary material

**Scopus**

( TITLE-ABS-KEY ( "School social climate" OR "School life" OR "Schoolwide climate" OR "School environment" OR "school climate" OR "school coexistence" OR "educational community" OR "classroom climate" OR "school connivance" ) AND TITLE-ABS-KEY ( scale OR measure OR measuring OR score OR rating OR survey OR questionnaire OR inventory OR index OR evaluation OR assess* OR instrument OR measurement OR detection OR diagnos* OR checklist OR psychometr* OR reliability OR validity ) AND TITLE-ABS-KEY ( argentina OR bolivia OR chile OR colombia OR "Costa Rica" OR cuba OR "Dominican Republic" OR ecuador OR "El Salvador" OR guatemala OR honduras OR mexico OR nicaragua OR panama OR paraguay OR peru OR "Puerto Rico" OR uruguay OR venezuela OR "Hispanic America" OR caribbean OR "Central America" OR "South America" OR "Latin America" OR "Spanish-speaking population" ) ) AND ( LIMIT-TO ( PUBYEAR , 2023 ) OR LIMIT-TO ( PUBYEAR , 2022 ) OR LIMIT-TO ( PUBYEAR , 2021 ) OR LIMIT-TO ( PUBYEAR , 2020 ) OR LIMIT-TO ( PUBYEAR , 2019 ) OR LIMIT-TO ( PUBYEAR , 2018 ) OR LIMIT-TO ( PUBYEAR , 2017 ) OR LIMIT-TO ( PUBYEAR , 2016 ) OR LIMIT-TO ( PUBYEAR , 2015 ) OR LIMIT-TO ( PUBYEAR , 2014 ) OR LIMIT-TO ( PUBYEAR , 2013 ) OR LIMIT-TO ( PUBYEAR , 2012 ) OR LIMIT-TO ( PUBYEAR , 2011 ) OR LIMIT-TO ( PUBYEAR , 2010 ) ).

**Web of Science and ScieLO**

"School social climate" OR "School life" OR "Schoolwide climate" OR "School environment" OR "school climate" OR "school coexistence" OR "educational community" OR "classroom climate" OR "school connivance" (Topic) AND scale OR measure OR measuring OR score OR rating OR survey OR questionnaire OR inventory OR index OR evaluation OR assess* OR instrument OR measurement OR detection OR diagnos* OR checklist OR psychometr* OR reliability OR validity (Topic) AND argentina OR bolivia OR chile OR colombia OR "Costa Rica" OR cuba OR "Dominican Republic" OR ecuador OR "El Salvador" OR guatemala OR honduras OR mexico OR nicaragua OR panama OR paraguay OR peru OR "Puerto Rico" OR uruguay OR venezuela OR "Hispanic America" OR caribbean OR "Central America" OR "South America" OR "Latin America" OR "Spanish-speaking population" (Topic) and 2023 or 2022 or 2021 or 2020 or 2019 or 2018 or 2017 or 2016 or 2015 or 2014 or 2013 or 2012 or 2011 or 2010 (Publication Years).

**Psycinfo***

tiab("School social climate" OR "school climate" OR "School life" OR "School-wide climate" OR "School environment" OR "school coexistence" OR "educational community" OR "classroom climate" OR "school connivance") AND tiab(Scale OR Measure OR Measuring OR Score OR Rating OR Survey OR Questionnaire OR Inventory OR Index OR Evaluation OR Assess* OR Instrument OR measurement OR detection OR diagnos* OR checklist OR psychometr* OR reliability OR validity) AND (Argentina OR Bolivia OR Chile OR Colombia OR "Costa Rica" OR Cuba OR "Dominican Republic" OR Ecuador OR "El Salvador" OR Guatemala OR Honduras OR Mexico OR Nicaragua OR Panama OR Paraguay OR Peru OR "Puerto Rico" OR Uruguay OR Venezuela OR "Hispanic America" OR Caribbean OR "Central America" OR "South America" OR "Latin America" OR "Spanish-speaking population")

* Limiting registrations after 01/01/2010
